# Dilated cardiomyopathy-mediated heart failure induces a unique skeletal muscle myopathy with inflammation

**DOI:** 10.1186/s13395-019-0189-y

**Published:** 2019-01-24

**Authors:** Taejeong Song, Palanikumar Manoharan, Douglas P. Millay, Sheryl E. Koch, Jack Rubinstein, Judith A. Heiny, Sakthivel Sadayappan

**Affiliations:** 1 Heart Lung Vascular Institute, Division of Cardiology, University of Cincinnati, Cincinnati, OH 45267 USA; 2 Department of Molecular Genetics, Biochemistry, and Microbiology, University of Cincinnati, Cincinnati, OH 45267 USA; 3 Division of Molecular Cardiovascular Biology, Cincinnati Children’s Hospital Medical Center, 240 Albert Sabin Way, Cincinnati, OH 45229 USA; 4 Department of Pediatrics, University of Cincinnati College of Medicine, Cincinnati, OH 45267 USA; 5 Department of Pharmacology and Systems Physiology, University of Cincinnati, Cincinnati, OH 45267 USA; 6 Department of Internal Medicine, Heart, Lung and Vascular Institute, Division of Cardiovascular Health and Sciences, College of Medicine, University of Cincinnati, 231 Albert Sabin Way, Cincinnati, OH 45267-0575 USA

**Keywords:** Heart failure, Dilated cardiomyopathy, Skeletal myopathy, Muscle contraction, Exercise

## Abstract

**Background:**

Skeletal muscle myopathy and exercise intolerance are diagnostic hallmarks of heart failure (HF). However, the molecular adaptations of skeletal muscles during dilated cardiomyopathy (DCM)-mediated HF are not completely understood.

**Methods:**

Skeletal muscle structure and function were compared in wild-type (WT) and cardiac myosin binding protein-C null mice (t/t), which develop DCM-induced HF. Cardiac function was examined by echocardiography. Exercise tolerance was measured using a graded maximum treadmill running test. Hindlimb muscle function was assessed in vivo from measurements of plantar flexor strength. Inflammatory status was evaluated from the expression of inflammatory markers and the presence of specific immune cell types in gastrocnemius muscles. Muscle regenerative capacityat days 3, 7, and 14 after eccentric contraction-induced injury was determined from the number of phenotypically new and adult fibers in the gastrocnemius, and functional recovery of plantar flexion torque.

**Results:**

t/t mice developed DCM-induced HF in association with profound exercise intolerance, consistent with previous reports. Compared to WT, t/t mouse hearts show significant hypertrophy of the atria and ventricles and reduced fractional shortening, both systolic and diastolic. In parallel, the skeletal muscles of t/t mice exhibit weakness and myopathy. Compared to WT, plantar flexor muscles of t/t null mice produce less peak isometric plantar torque (Po), develop torque more slowly (+ dF/dt), and relax more slowly (− dF/dt, longer half-relaxation times,1/2RT). Gastrocnemius muscles of t/t mice have a greater number of fibers with smaller diameters and central nuclei. Oxidative fibers, both type I and type IIa, show significantly smaller cross-sectional areas and more central nuclei. These fiber phenotypes suggest ongoing repair and regeneration under homeostatic conditions. In addition, the ability of muscles to recover and regenerate after acute injury is impaired in t/t mice.

**Conclusions:**

Our studies concluded that DCM-induced HF induces a unique skeletal myopathy characterized by decreased muscle strength, atrophy of oxidative fiber types, ongoing inflammation and damage under homeostasis, and impaired regeneration after acute muscle injury. Furthermore, this unique myopathy in DCM-induced HF likely contributes to and exacerbates exercise intolerance. Therefore, efforts to develop therapeutic interventions to treat skeletal myopathy during DCM-induced HF should be considered.

**Electronic supplementary material:**

The online version of this article (10.1186/s13395-019-0189-y) contains supplementary material, which is available to authorized users.

## Background

Heart failure (HF) is a leading cause of death worldwide. HF compromises not only cardiac muscle function and structure but also peripheral tissues, especially the skeletal muscles [1]. Maintaining skeletal muscle mass and functional capacity is essential for HF patients to maintain independence and benefit from therapies. Skeletal muscle maladaptations in HF cause a significant loss of physical capacity and worsen prognosis [2]. Notably, muscle wasting in HF is a strong independent risk factor for mortality [3].

Intriguingly, skeletal muscle maladaptations in HF do not correlate with the severity of HF or reduced cardiac output and oxygen delivery [4, 5]. This finding suggests the existence of skeletal muscle-specific impairments. However, beyond consistent reports of reduced exercise tolerance and fatigue resistance, the skeletal muscle maladaptations in HF remain incompletely defined and their underlying mechanisms are poorly understood.

Skeletal muscle weakness and reduced exercise capacity are common debilitating symptoms in patients with heart failure. These impairments are often associated with decreased mass, fiber type switching, mitochondrial dysfunction, and anabolic/catabolic signal imbalances [6–8]. However, some recent studies report the preservation of muscle mass, contractile function, and muscle fiber types in HF [9, 10]. For example, in patients with chronic heart failure (CHF), maximum voluntary isometric force of quadriceps muscle and specific tension of skinned vastus lateralis biopsies are reduced [11, 12]. On the other hand, one study of human patients with CHF found no significant impairment of peak isokinetic torque during knee extension [4]. Soleus muscles of a rat model of CHF maintain maximum tetanic force [13]. These mixed results may be attributed to the different models of HF used in different studies, as well as heterogeneity and comorbidity in the patient populations studied.

In animal studies, invasive surgery (e.g., coronary artery ligation) or toxin injection (e.g., monocrotaline) are commonly used to induce HF via sudden myocardial infarction and pulmonary hypertension. However, HF progression in humans with familial dilated cardiomyopathy (DCM), identified in up to 35% of DCM cases [14], differs from these models in important ways. Systemic, chronic inflammation in association with elevated proinflammatory cytokines has been widely reported in human HF patients and in animal models of HF [15, 16]. Local ischemia and peripheral vasoconstriction also commonly occur and both are known to induce muscle damage and impair the intrinsic regenerative capacity of skeletal muscles [17, 18]. However, the skeletal muscle-specific impairments in DCM-induced HF in mice have not been previously examined.

Ablation of cMyBP-C expression in cMyBP-C null (t/t) mice induces a DCM-induced HF that closely resembles DCM-induced HF in humans [19]. MyBP-C is a cardiac-specific sarcomeric protein that crosslinks thick (myosin) and thin (actin) filaments at specialized locations in the A band. The mutation in this model eliminates expression of cMyBP-C protein. Because cMyBP-C is expressed only in the heart, it is essentially a cardiac muscle-targeted model of HF. The cMyBP-C-encoding gene, *MYBPC3*, is the most frequently mutated gene in human cardiac myopathy (HCM). Mutations in *MYBPC3* cause HCM and DCM and both progress to HF [17].

The present study compared skeletal muscle contractile function, structural adaptations, inflammatory status, and regenerative capacity in WT and t/t mice. Our results show that DCM-induced HF in mice is associated with a unique skeletal muscle myopathy characterized by decreased muscle strength, atrophy and loss of oxidative fiber types, ongoing muscle inflammation and damage, and impaired regeneration of damaged muscle. This novel myopathy likely contributes to and exacerbates exercise intolerance in DCM-induced HF.

## Methods

### Animals

Adult homozygous cMyBP-C null (t/t) and nontransgenic FVB/N wild-type (WT) mice, aged 4 to 8 months, were used. In the t/t mouse, a mutation in the C-terminal region of cMyBP-C, which mediates myosin anchoring to actin, leads to a cMyBP-C null heart and DCM-induced HF [19]. All animal protocols were approved by the Institutional Animal Care and Use Committee at the University of Cincinnati. All animals were anesthetized via 1.5–2.0% isoflurane inhalation on a heated stage and were sacrificed by cervical dislocation.

### Cardiac phenotype and function

Cardiac function was evaluated in anesthetized mice using a Vevo 2100 imaging system (VisualSonics**,** Toronto, Canada). M-mode echocardiography imaging was performed at a parasternal long axis [15]. Fractional shortening (%FS) and left ventricular internal diameter during end-systole (LVID,s) and end-diastole (LVID,d) were analyzed using Vevo Strain software (Vevo 2100, v1.6). The hearts were excised immediately following the measurements, embedded in O.C.T compound, and frozen in liquid nitrogen-cooled isopentane. The frozen hearts were sectioned coronally into 10-μm thick sections using a cryostat microtome at − 15 °C and then stained with hematoxylin and eosin for histological analysis.

### Exercise tolerance test

Exercise capacity was examined by a graded maximum running test performed on a computerized mouse treadmill (Omnitech Electronics, Columbus, OH, USA). Prior to the test, mice were acclimated to treadmill running by three practice sessions conducted over 3 days. Practice sessions consisted of 10-min running trials at 4 to 10 m/min. On the day of testing, mice were placed on the treadmill and kept calm for 5 min. Then, they ran until exhaustion at the following speeds, angles, and durations: (6 m/min, 0°, 2 min), (9 m/min, 5°, 2 min), (12 m/min, 10°, 2 min), (15 m/min, 15°, 2 min), (18, 21, 23 m/m, 15°, 1 min), and addition of 1 m/min at 15° for every 1 min thereafter. Exhaustion (cessation of the test) was declared when a mouse sat on the shock grid for more than 5 s.

### In vivo hindlimb muscle contractile function

Contractile function of plantar flexor muscles was evaluated in vivo from measurements of isometric plantar flexion torque. An anesthetized mouse was placed on a heated platform in supine position with a radiant lamp placed above. One knee was clamped in a 90° joint angle, and the foot was secured to the pedal (2 cm lever length) of a dual-mode servometer (300C-LR: Aurora Scientific, Aurora, ON, Canada). To stimulate the plantar flexors, a pair of needle electrodes connected to an electrical stimulator (701C, Aurora Scientific, Aurora, ON, Canada) was inserted percutaneously into the posterior side of the knee near the tibial nerve. Initially, isometric twitch torque (Pt) was measured in response to 0.2 msec pulses at 50 mA and used to determine optimal leg position. Next, peak isometric tetanic torque (Po) was measured at the optimal muscle length in response to tetanic stimulation (0.2 msec pulses applied at 50 to 150 Hz frequency for 350 msec duration at a repetition rate of 2 min). Force output was digitized and analyzed using Dynamic Muscle Control (DMC v5.5) and Dynamic Muscle Analysis (DMA v5.3), respectively (Aurora Scientific, Aurora, ON, Canada).

### Eccentric contraction-induced muscle injury

The plantar flexors were subjected to repeated eccentric muscle contractions (ECC) which were induced by forced dorsiflexion while plantar flexors generated maximum tetanic torque at 150 Hz for 350 msec. ECC contractions were repeated 100 times every 5 s, and the angle of dorsiflexion was set to 14^o^ at the speed of 0.7°/10 msec (representative ECC force-time graph in Fig. 6a). Recovery of torque (Po) was evaluated at 3, 7, and 14 days after ECC injury. Then, mice were sacrificed, and hindlimb muscles were excised and weighed. The gastrocnemius (GAS) muscles were separated into medial (MG) and lateral (LG) parts, embedded in OCT, frozen in liquid nitrogen-cooled isopentane, and stored at − 80 °C until analysis.

### Immunohistological analysis

The proximal belly region of LG muscles (*n* = 3 males in each group) was cross-sectioned at 10 μm thickness with a cryostat (CM1900, Leica Microsystems; Wetzlar, Germany) at − 15 °C. A slide was prepared, stained with H&E, and examined under an inverted microscope (Olympus IX73: Olympus, Tokyo, Japan). Images were acquired digitally (Olympus DP73 camera: Olympus, Tokyo, Japan) and analyzed using ImageJ software (NIH) to determine fiber cross-sectional areas (CSA) and number of centrally located nuclei (CN). To determine fiber types, cross-sectioned LG samples were fixed with cold acetone for 5 min and permeabilized with 0.2% Triton X-100 in PBS for 10 min. Slides were blocked in 5% Fab fragment (Jackson 111–007-003) for 1 h and incubated overnight at 4C with primary antibodies for MHC isoforms (MHC type I, BA-D5; MHC type IIa, SC-71; MHC type IIb, BF-F3; Developmental Studies Hybridoma Bank, University of Iowa), embryonic myosin heavy chain (eMHC, HPA021808, Sigma-Aldrich), Pax7 (20570–1-AP, Proteintech), dystrophin (MA1–26837, Invitrogen), and laminin (L9393, Sigma-Aldrich). Thereafter, the slides were incubated for 1 h in secondary antibody (Alexa Fluor, Invitrogen) at room temperature, mounted with anti-fade mounting media (P36930 or S36942, Invitrogen), imaged at 10X or 20X lens using inverted microscope (Leica DMi8 or Olympus IX73), and analyzed in a blinded manner using ImageJ or Las X software (Leica).

### RNA isolation and real-time quantitative PCR (RT-qPCR)

Total RNA was isolated from GAS muscle and macrophages using the mir Vana™ miRNA Isolation Kit (Invitrogen). cDNA was synthesized using the Superscript IV reverse-transcription system (Invitrogen, # 18091050), and genomic DNA was eliminated using EZDNASE (Invitrogen, # 11766051). mRNA expression was quantified using gene-specific TaqMan primers and probes (Applied Biosystems), following the manufacturer’s protocol, and normalized to 18sRNA levels. RT-qPCR was performed using an ABI7300 real-time PCR system (Applied Biosystems) and Taqman universal master mix (Applied Biosystems, #4440044). Relative gene expression was calculated using the ΔΔ*C*_*t*_ method. All reactions were carried out in triplicate.

### Cell isolation and flow cytometry

Flow cytometry was performed using a suspension of single cells obtained from GAS. Briefly, the muscle was placed in warmed DMEM (Life Technologies) and chopped into small pieces, followed by enzymatic dissociation using a skeletal muscle dissociation kit (Miltenyi Biotec, #130-098-305). The dissociated sample was filtered through a 70-μm cell strainer (Fisher Brand). Red blood cells (RBCs) were lysed with RBC Lysing buffer (eBioscience, #00-4300-54) at room temperature for 10 min. After lysis, cells were centrifuged, and the pellet was resuspended in flow cytometry buffer (eBioscience, # 00-4222-26). The samples were blocked with CD16/32 for 10 min at 4 °C. For flow cytometry analysis of monocytes and macrophages, the cells were stained with Ly6C-APC (eBioscience), CD11B-FITC (BioLegend), and F4/80-PECy7 (BioLegend) for 1 h at 4 °C. Sytox blue (Invitrogen) was used to exclude dead cells and debris, and unstained controls were used to set gates. Samples of 30,000 cells per run were analyzed in the flow cytometer (LSR Fortessa, BD Bioscience), and the resulting data were analyzed using FlowJo software (FlowJo, LLC).

### Statistical analysis

Data are presented as mean ± SE. Student’s *t* test was used for group comparisons (WT vs. t/t). Statistical analysis for comparing within and between group variables was performed by two-way ANOVA with Bonferroni post hoc test using GraphPad Prism 7.04 software. Statistical significance is defined as *P* < 0.05.

## Results

### HF reduces exercise capacity and muscle strength without change in muscle mass

**Fig. 1:**
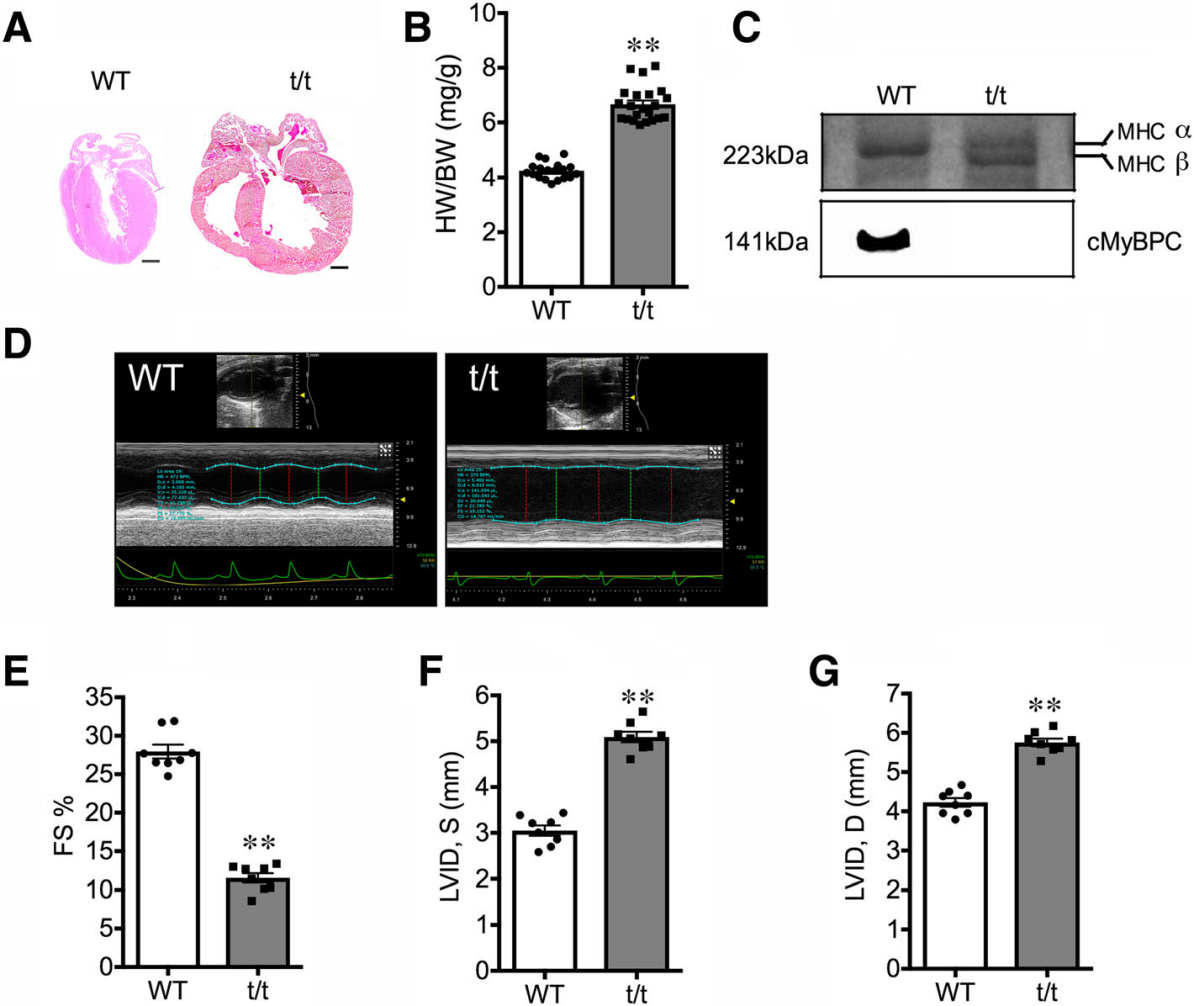
Severe DCM phenotypes and impaired cardiac function in t/t hearts. **a** Slide of coronally sectioned heart shows dilated atrium and ventricles in t/t heart. **b** Dilated cardiomyopathy (DCM) was evidenced by a significant increase in heart-to-body weight ratio (*n* = 19 and 22 in WT and t/t respectively). **c** Myosin heavy chain (MHC) isoform shifted from type *α* to *β*, and no cMyBP-C protein was detected in t/t heart sample (bottom). Cardiac function was examined by M-mode echocardiography. **d** Representative short-axis M-mode echocardiographic images of WT and t/t hearts. In t/t, fractional shortening (**e**) decreased, but left ventricle internal diameters during end-systole (**f**) and end-diastole (**g**) increased compared with those of WT. Scale bar: 1 mm and **P < 0.01. *n* = 8 in each group

The t/t mice developed diagnostic features of DCM-induced HF (Fig. 1), as described previously [15, 20] . Compared to WT, the atria and ventricles of t/t hearts were visibly enlarged (Fig. 1a), heart-to-body weight ratio increased by 57% (Fig. 1b), and MHCs switched from *α* to *β* forms (Fig. 1c). On echocardiography (Fig. 1d), fractional shortening (FS %) decreased by 59% compared to WT (Fig. 1e, *P* < 0.01). Left ventricle internal diameters increased by 2.04 and 1.53 mm during systole (Fig. 1f) and diastole, respectfully (Fig. 1g, *P* < 0.01). These cardiac phenotypes appeared 2 weeks after birth and progressively worsened with age (data not shown). In addition, t/t mice developed significant exercise intolerance. When tested at age 4 to 5 months for their ability to perform treadmill running at incremental speeds and angles, t/t mice ran for significantly shorter durations (− 24%) compared to WT (Additional file 1: Figure S1A, *P* = 0.032). The decreased exercise tolerance did not result from cardiac cachexia-induced muscle wasting. Average body mass (28.86 ± 0.55 g vs. 28.29 ± 0.70 g; t/t and WT, respectively) and the mass of major hindlimb muscles (soleus, gastrocnemius, plantaris, soleus, tibialis and EDL) measured at 8 months of age did not differ between genotypes (Additional file 1: Figure S1B-F).

**Fig. 2:**
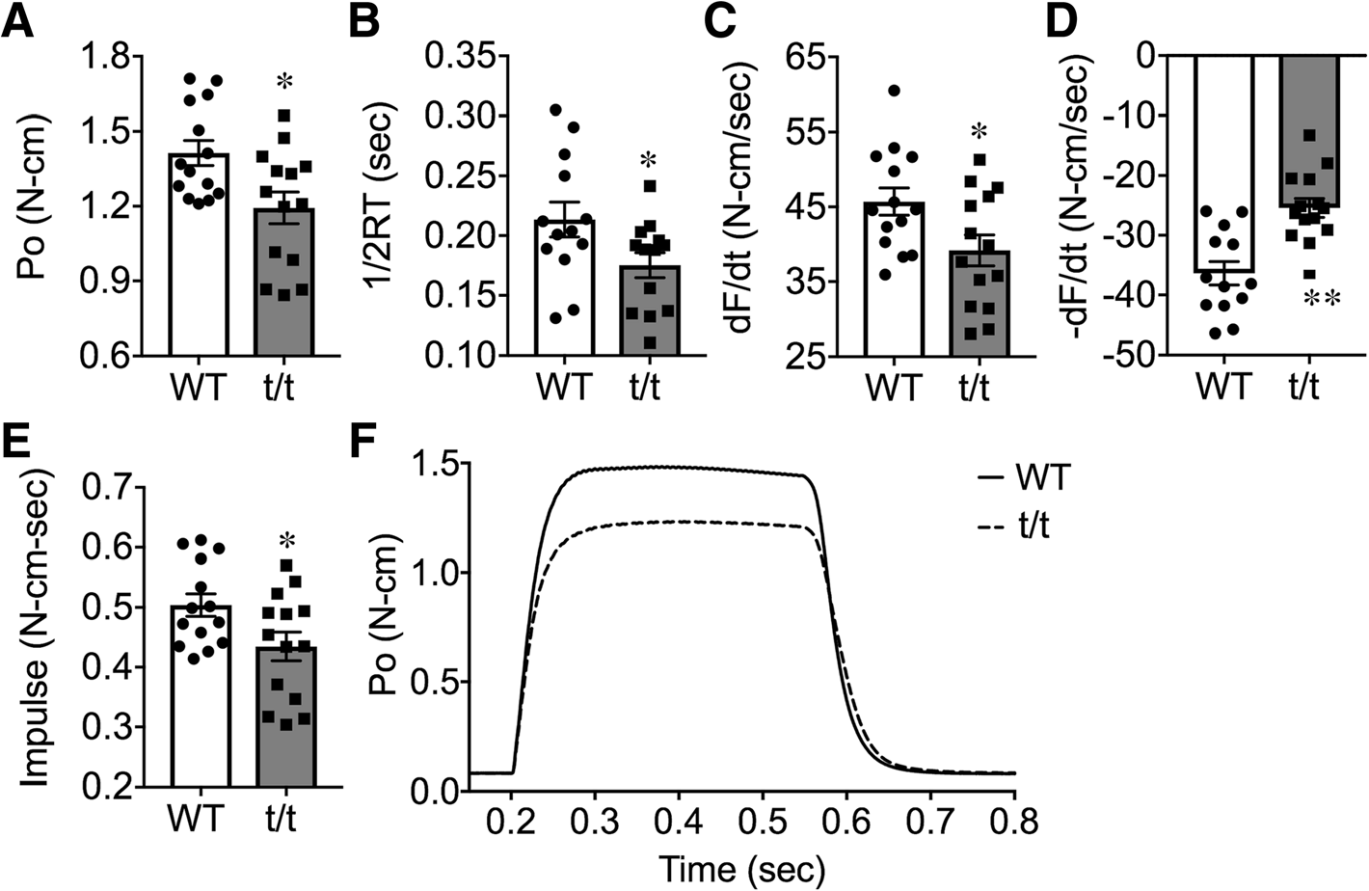
Decrease of maximum muscle strength and contractile function in t/t compared to WT. **a** Po, peak isometric tetanic torque produced in vivo by plantar flexor muscles fixed at optimal leg position. Tetanic torque was evoked by applying supramaximal stimuli to the distal portion of the tibial nerve (0.2 msec duration at 150 Hz for 350 msec). **b** 1/2RT, half-relaxation time. **c** dF/dt, peak rate of torque development. **d** dF/dt, peak rate of torque relaxation. **e** impulse (integration of torque with time). **f** Po(t) in t/t and WT. *P < 0.05 and **P < 0.01 *n* = 13–14 in each group

The skeletal muscles of t/t mice showed significantly reduced contractility (Fig. 2). Peak isometric tetanic torque (Po, N-cm) produced by plantar flexor muscles, measured in vivo at optimal leg position, was significantly reduced in t/t compared to WT (− 16%, Fig. 2a, *P* = 0.012). Rates of torque development (dF/dt) and relaxation (dF/dt) also decreased (Fig. 2c and d -14%, *P* = 0.036 and − 30%, *P* = 0.0002, respectively). Half-relaxation time (1/2RT) (− 18%) and impulse (− 14%) were reduced in t/t mice compared to WT (Fig. 2b and e, *P* = 0.045 and 0.032). These impairments occurred without change in peak twitch torque (Pt) elicited by a single supramaximal stimulus (Additional file 1: Table S1). Overall, plantar flexor muscles of t/t mice contracted and relaxed slower and produced less torque than WT (Fig. 2f). Reduced contractility was further evident in the torque-frequency response of plantar flexor muscles (Additional file 1: Figure S2A, *P* < 0.05). Evoked torque increased with stimulation frequency, as expected; however, t/t muscles reached a maximum tetanic torque at 100 Hz, while WT mice were able to increase torque up to 125 Hz. Differences in the kinetics of torque production were also greatest at the highest frequencies (Additional file 1: Figure S2B, *P* < 0.05 and 2C, *P* < 0.05). At stimulation frequencies above 100 Hz, the rate of force relaxation (− dF/dt) was significantly less in t/t compared to WT (Additional file 1: Figure S2D, *P* < 0.05 and *P* < 0.01). The decreased contractility was not due to submaximal electrical stimulation (Additional file 1: Figure S2). Collectively, these results suggest that DCM-induced HF is associated with significant impairments in skeletal muscle force generation.

### HF alters skeletal muscle structure

**Fig. 3:**
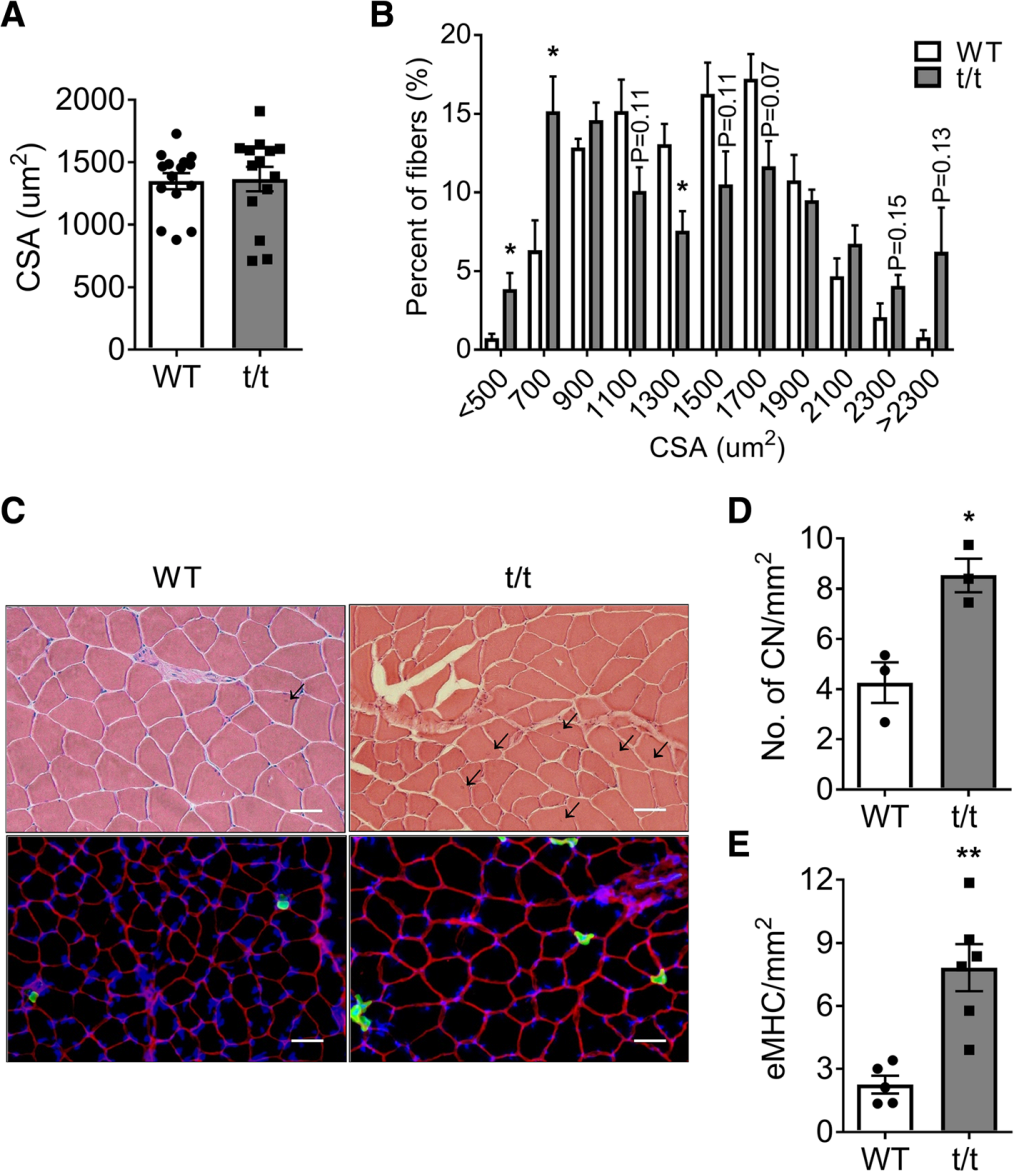
Histological maladaptations in t/t LG muscle. Average cross-sectional area (CSA) of individual muscle fibers (**a**) and their size distribution (**b**) were evaluated in over 500 fibers from each sample. **c** H&E staining (top) and immunohistology (eMHC; green, dystrophin; red, and DAPI; blue, bottom) of cross-sectioned LG muscle. **d**, **e** Numbers of central nuclei (CN, **d**) and eMHC+ fibers (**e**) were counted on whole muscle cross-section and normalized by mm^2^ area. Arrowhead indicates CN (scale bar: 50 um). *P < 0.05 and **P < 0.01. *n* = 3 muscle samples of each genotype. An average of 521 ± 11 fibers from five different areas of each slide were used for analysis

**Fig. 4:**
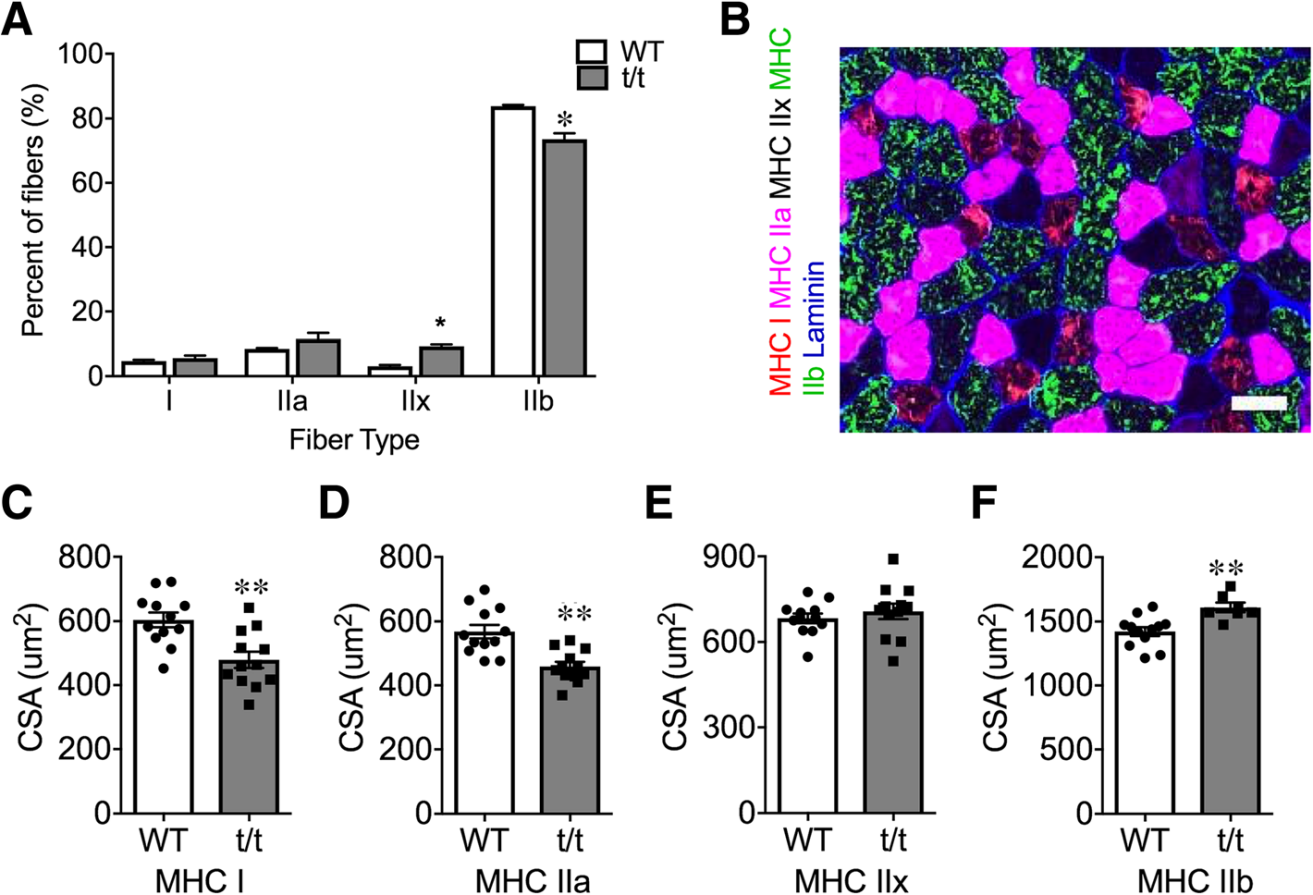
Preserved fiber type distribution, but atrophied oxidative muscle fibers. **a** Fiber type distribution in LG determined from cross sections labeled with MHC isoform-specific antibodies. **b** Representative image of immunostained LG with MHCs and Laminin antibodies. Scale bar: 50 μm. **c**–**f**, CSA of each fiber type in WT and t/t. Number of muscle samples = 3 in each group. *P < 0.05 and **P < 0.01

To further define the myopathy in t/t mice, we compared skeletal muscle morphology in t/t and WT mice. The average fiber size in the LG muscle determined from measurement of fiber CSA on H&E stained sections was not significantly different between genotypes (Fig. 3a). However, the distribution of muscle CSA was skewed (Fig. 3b), with t/t muscles having a greater number of small fibers (< 700 μm^2^, + 12%, *P* = 0.037) and a modestly increased number of large fibers (> 2000 μm^2^, + 9%, *P* = 0.08). This result suggests that DCM-induced HF may cause muscle atrophy and possibly hypertrophy in distinct subpopulations of fibers. To investigate this possibility, we compared the size and distribution of fiber types using myosin isoform-specific antibodies (Fig. 4). In WT LG, type IIb fibers accounted for 83.7% of the total, and, proportionally, type I, IIa, and IIx fibers accounted for 4.7, 8.5, and 3.1%, respectively. In t/t LG, fiber types I and IIa comprised 5.7 and 11.5% of the total values that were not significantly different from WT. However, the percentage of type IIx fibers increased to 9.3% and type IIb fibers decreased to 73.6% (Fig. 4a, *P* = 0.0013 and 0.007). Compared to WT, the average CSA of type I and IIa fibers decreased by 21 and 19%, respectively (Fig. 4c and d, *P* = 0.0015 and 0.0003), while the average CSA of type IIb fibers increased 13% (Fig. 4f, *P* = 0.0027). This result excludes the possibility that a slow-to-fast fiber type remodeling occurred in t/t muscles. Instead, it shows a selective decrease in the number and size of oxidative fiber types, both slow type I and fast oxidative type IIa, in association with hypertrophy of fast glycolytic type IIb fibers and increased number of type IIx fibers. Additionally, LG muscles of t/t mice have twice the number of fibers with central nuclei (CN) compared to WT (8.53 ± 0.67 vs. 4.26 ± 0.81; Fig. 3d, *P* = 0.015). The numbers of fibers expressing embryonic myosin heavy chain (eMHC) is also significantly greater in t/t muscle (7.83 vs. 2.26 in WT; Fig. 3e, *P* = 0.002). The presence of fibers with CN and eMHC, markers of immature newly forming fibers, suggests ongoing regeneration in t/t muscles under homeostasis.

### HF enhances inflammatory mediators and suppresses myogenic signals in skeletal muscle

**Fig. 5:**
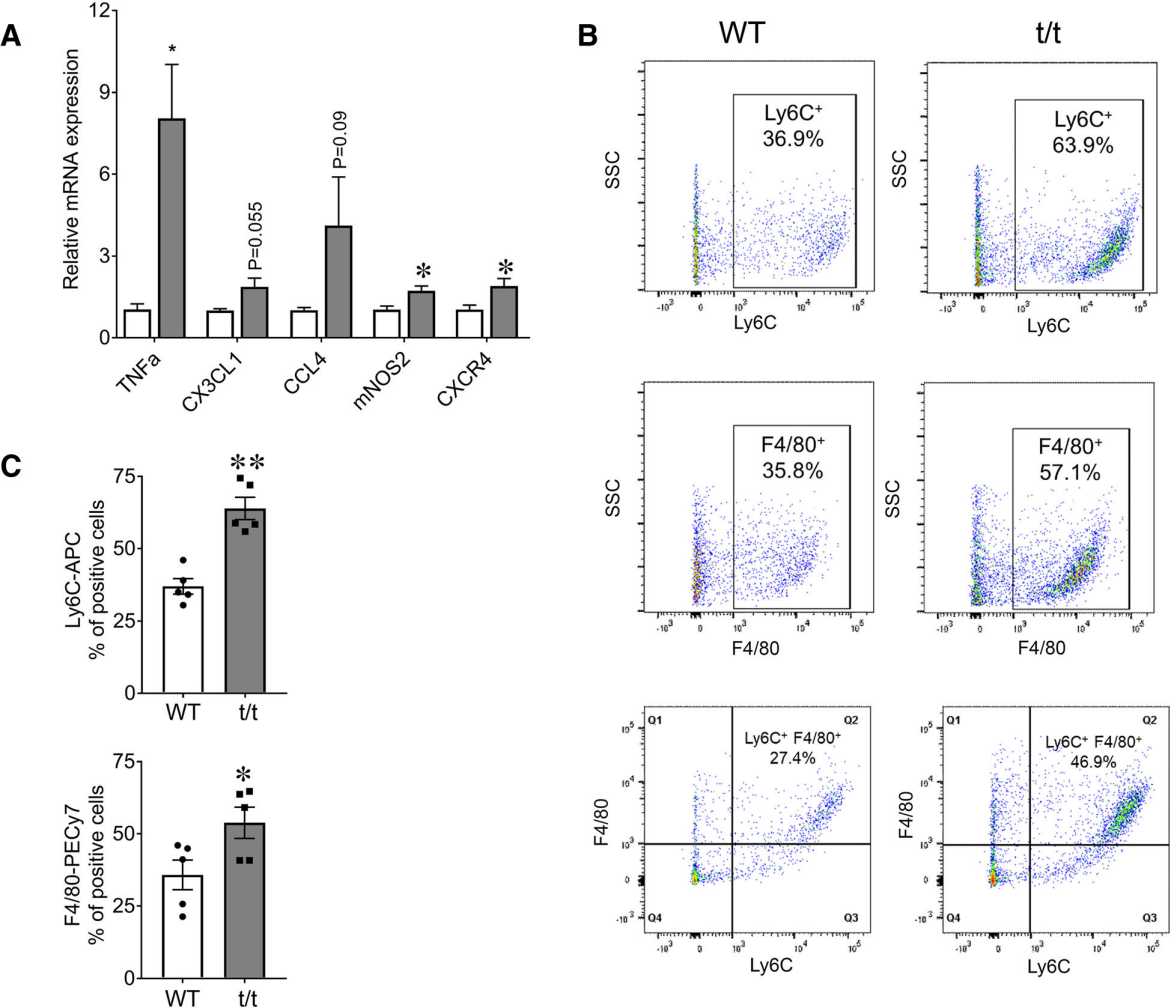
Elevated expression of inflammatory markers and cells in t/t GAS. **a** mRNA levels of inflammatory mediators and chemokine receptors measured by RT-qPCR in GAS muscles showed increased expression in t/t compared to WT mice. Representative flow cytometry analysis of monocytes showed Ly6C^+^- and F4/80^+^-gated cells from GAS muscle of t/t and WT mice (**b**). The percentage of Ly6C^+^ and F4/80^+^ cells (**c**) was significantly greater in GAS of the t/t mice (Ly6C^+^ − 36.9% in WT vs. 63.9% in t/t mice (*n* = 5, **P < 0.001); F4/80^+^ − 35.8% in WT vs. 57.1% in t/t mice (*n* = 4, *P < 0.02)). Ly6C^+^ cells were further sorted by F4/80 gating to determine if the tissue-specific macrophages were positive for the inflammatory marker. t/t mice showed an increase in the double positive population (as shown in Q2), indicating a greater number of intramuscular inflammatory macrophages (**b**, bottom)

Previous studies from our lab have shown that t/t mice exhibit robust systemic and local inflammatory responses and that their hearts show cellular damage characteristic of chronic HF [15]. It is not known whether local inflammation and cellular damage also affect the skeletal muscles of mice with DCM-induced HF. To investigate this question, we examined the expression of inflammatory mediators in GAS muscles from t/t and WT mice (Fig. 5a). TNFα, mNOS2, and the chemokine receptor CXCR4 were significantly increased in t/t muscles compared to WT (TNF-α + 772%, mNOS2 + 68%, and CXCR4 + 83%; Fig. 5a, *P* = 0.024, 0.025, and 0.037). TNF-α and mNOS2 are proinflammatory mediators that originate from both infiltrating immune cells and damaged muscle fibers [21–23]. The abundance of CXCR4, a chemokine receptor for ligand SDF1 (CXCL12), indicates inflammatory myopathy conditions [24]. Upregulation of these inflammatory signals suggests ongoing inflammation and cellular damage.

Damaged skeletal muscles recruit inflammatory committed Ly6C^+^ monocytes (one of the two main populations of circulating monocytes in mice) from the circulation as an initial response to damage [25]. Ly6C^+^ monocytes are sparse in uninjured skeletal muscles, and their presence indicates ongoing damage. Within the damaged muscle, recruited Ly6C^+^ monocytes mature into inflammatory Ly6C^+^ macrophages which express the tissue macrophage marker F4/80 [25]. GAS muscles of t/t mice have greater numbers of Ly6C^+^ monocytes and Ly6C^+^F4/80^+^ macrophages compared to WT (Fig. 5c; top, *P* = 0.0012). The percentage of cells expressing F4/80^+^ as well as dual-labeled Ly6C^+^ F4/80^+^ cells, was significantly greater in t/t GAS (Fig. 5c; bottom and b; bottom, *P* = 0.047), while populations expressing CD11B+, a general marker of all inflammatory monocytes, both immature and activated, were not significantly different (data not shown). In dual-labeled Ly6C^+^ F4/80^+^ cells represent monocyte-derived macrophages that matured from infiltrating Ly6C^+^ monocytes. In increased numbers of dual-labeled Ly6C^+^ F4/80^+^ monocyte-derived macrophages are also present in cardiac muscle of t/t mice [15]. Altogether, t/t GAS muscles showed elevated expression of proinflammatory mediators in association with increased infiltration of inflammatory immune cells and a greater number of monocyte-derived inflammatory macrophages. Their persistent presence suggests ongoing damage.

Collectively, the enhanced expression of inflammatory mediators, in association with elevated numbers of inflammatory monocytes and macrophages, suggest that regeneration is not able to keep pace with muscle damage under homeostasis.

### HF reduces skeletal muscle repair and regeneration capacity

**Fig. 6:**
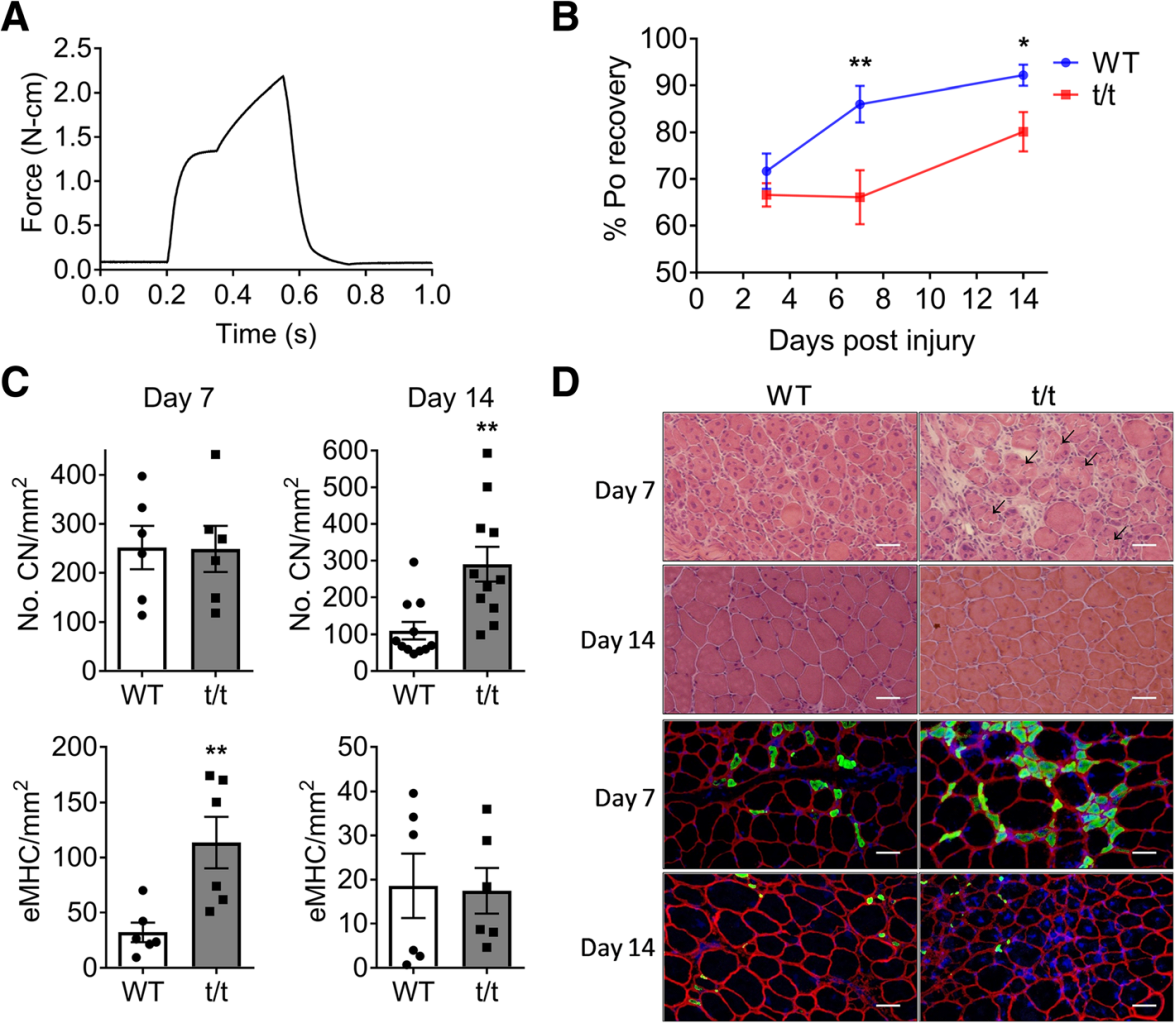
Impaired functional recovery and muscle regenerative capacity after injury. **a** Representative Po graph during eccentric muscle contraction (ECC). **b** Po recovery at 3, 7, and 14 days after ECC injury. **c** Numbers of CN (top) and eMHC+ fibers (bottom) were counted on injured LG at 7 (left) and 14 days (right) post-injury and compared between t/t and WT groups. **d** Cross-sectioned images of injured LG stained by H&E (top) and immunostained with antibodies (eMHC; green, dystrophin; red, and DAPI; blue, bottom). Scale bar:50 μm. Arrowhead indicates necrotic fiber. Number of muscle samples = 3 in each group. **P < 0.01 vs. t/t; *P < 0.05 vs. day 3

Above results suggest that the ability to repair and regenerate damaged tissue may be impaired in muscles of t/t mice. To examine this possibility, we evaluated the ability of plantar flexor muscles to repair after acute injury induced by ECC muscle contractions (Fig. 6b and the “Methods” section). Skeletal muscle regeneration after acute injury requires a programmed immune response that is essential for proper repair [26]. The ongoing systemic and local inflammation in t/t mice may interfere with this innate program, as proposed [18]. At day 3 post-injury, plantar flexor torque was reduced by − 31% and − 34% in WT and t/t mice, respectively (Fig. 6b), confirming that ECC caused equivalent muscle damage in both genotypes. However, at day 7 post-injury, WT plantar flexors had recovered 86% of Po, whereas t/t plantar flexors had not improved from day 3 (Fig. 6b, *P* = 0.009). By day 14, WT mice regained 95% of initial Po, while t/t mice regained only 83% (Fig. 6b). At 7 days post-injury, fibers with CN, which indicate ongoing myogenesis, were present in both WT and t/t mice (126/mm^2^ and 124/mm^2^, respectively; Fig. 6c, top). However, at day 14 post-injury, the number of fibers with CN remained significantly elevated in t/t compared to WT mice (145/mm^2^ vs. 55/mm^2^) (Fig. 6c, top), indicating prolonged muscle degeneration. The muscles of t/t mice also showed significantly more necrotic fibers at 7 days post-injury (16.04/mm^2^ vs. 4.49/mm^2^ in WT, *P* = 0.008, Fig. 6d, arrowhead). Impairment of recovery after injury in t/t muscle is not due to a decrease in satellite cell number, which was not different in WT and t/t (Additional file 1: Figure S4). In addition, the number of eMHC-positive fibers, another marker of ongoing muscle regeneration, was significantly increased in t/t at 7 days post-injury compared to WT (114/mm^2^ vs. 32mm^2^, Fig. 6c, bottom, *P* = 0.009). At 14 days post-injury, the number of muscle fibers expressing eMHC decreased in both groups (18.6/mm^2^ and 17.4mm^2^ in WT and t/t, respectively, Fig. 6c, bottom). Together, these results indicate that recovery of structure and function after injury is delayed and/or incomplete in skeletal muscles of t/t compared to WT mice.

## Discussion

This study examined skeletal muscle adaptations in a mouse model of DCM-induced HF [19]. MyBP-C null mice carry a mutation in the *MYBPC3* gene that ablates cMyBP-C protein expression in cardiac muscle cells. *MYBPC3*, a cardiac-specific myosin binding protein, is one of the most frequently mutated genes in human cardiac myopathy [27]. Mutations in the *MYBPC3* gene in mice cause hypertrophic cardiac myopathy and DCM that progress to HF [17]. Mice which lack MyBP-C protein develop a cardiomyopathy that closely resembles DCM-induced HF in humans [15, 19]. MyBP-C null mice show hypertrophy of the atria and ventricles, increased heart-to-body weight ratio, reduced fractional shortening with increased left ventricular diameter on echocardiography during both systole and diastole, and exercise intolerance on treadmill running tests. The reduced exercise capacity does not result from cachexia secondary to HF; body mass and the average fiber CSAs of defined GAS muscles are unchanged compared to age-matched controls.

Skeletal muscle weakness and exercise intolerance are common and debilitating symptoms in patients with HF. Intriguingly, the skeletal muscle maladaptations do not correlate with the severity of HF or reductions in cardiac output and oxygen delivery [4, 5, 28], suggesting the existence of skeletal muscle-specific adaptations. However, skeletal muscle adaptations in HF and their underlying mechanisms remain poorly understood [11, 12].

This study identifies a unique set of adaptations in peripheral skeletal muscles of mice with DCM-induced HF. When stimulated repetitively to produce sustained torque, the plantar flexor muscles produce significantly less maximum tetanic torque with slowed rates of onset and relaxation. The greatest difference in tetanic torque was seen at the highest stimulation frequencies. This pattern could suggest a greater impairment of slow compared to fast muscle types, as proposed [28, 29] because force output by slow muscles saturates at lower stimulus frequencies. However, slow-to-fast fiber remodeling of skeletal muscles was not observed in this model. Rather, t/t muscles showed selective atrophy and decreased content of oxidative fiber types, both slow (MHC type I) and fast (MHC type IIa), together with modest hypertrophy of glycolytic fibers (MHC type IIb) and mild fiber type switching from type IIb to type IIx. These fiber type changes could be due, in part, to the greater number of regenerating fibers in t/t*.* Regenerating fibers are incompletely differentiated and undergo a transition to type IIb fibers. The selective atrophy and loss of oxidative fiber types may contribute to the decreased tetanic torque with exercise intolerance. Type I fibers are required to maintain tetanic force, and type IIa fibers are needed to sustain aerobic exercise, such as running. The loss and atrophy of oxidative fiber types may be a consequence of reduced oxygen delivery related to peripheral vasoconstriction and local ischemia [29].

DCM-induced HF in t/t mice is associated with local and systemic inflammation under homeostasis [15]. Proinflammatory macrophages are elevated in the heart, and proinflammatory cytokine IL-1 levels are elevated in blood [15]. The inflammatory status of skeletal muscles in this model is not known. Here, we show that skeletal muscles of t/t mice have significant local inflammation, suggesting ongoing damage. GAS muscles of t/t mice show elevated expression of the inflammatory mediators TNF-a, mNOS2, and CXCR4. In addition, they have elevated numbers of inflammatory immune cells recruited from the circulation (Ly6C^+^ monocytes) and intramuscular inflammatory monocyte-derived macrophages, namely, Ly6C^+^ and F4/80^+^. This macrophage phenotype is also found in cardiac muscle of t/t mice [15]. In mice, damaged skeletal muscles recruit Ly6C^+^ monocytes immediately after injury [25], and these mature within the muscle into inflammatory monocyte-derived macrophages. The presence of these inflammatory mediators and immune cell types in GAS muscles indicates ongoing damage with compensatory repair and regeneration under homeostasis.

TNF-α is produced by inflammatory MPs, and it increases transiently in injured muscles as part of the normal repair and regeneration process [22, 30]. However, chronic elevation of TNF-α under homeostasis suggests prolonged inflammation that does not resolve. Chronic exposure to TNF-α delays muscle regeneration, causes muscle wasting [31], and inhibits myoblast differentiation by reducing myogenin expression [32]. Elevated levels of TNF-α and inflammatory immune cells in t/t mice may interact in a positive feedback manner to exacerbate skeletal muscle damage, as well as contribute to the fiber necrosis seen in t/t LG at 7 days post-injury. Chronically elevated TNF-α levels may also impede differentiation of myogenic cells [33] and contribute to the sustained presence of immature myofibers with CN.

Ongoing inflammation and fiber damage trigger an innate program of regeneration as the muscle tries to replace damaged fibers. CN are present at an early stage of new fiber formation. The presence of CN indicates ongoing regeneration; however, their persistence suggests that regeneration is not completed and that not all fibers progress to multinucleated adult phenotypes with peripheral nuclei. Decreased levels of the myokines MYOG (myogenin) and MEG3 (Additional file 1: Figure S3C), which are required for the growth and differentiation of newly forming myofibers, likely contribute to incomplete regeneration in t/t mice [34]. Interestingly, MYOG expression is also decreased in cachexia associated with HF [35]. Collectively, these findings in t/t mice suggest both ongoing muscle damage and impaired regeneration under homeostasis.

An impaired ability to regenerate damaged fibers was also seen in t/t muscles after acute injury. WT skeletal muscles have a robust capacity to regenerate damaged tissue. However, muscles of t/t show delayed and incomplete recovery of torque after acute damage, indicating incomplete regeneration. The delayed recovery of force is associated with elevated inflammatory signals and cells, elevated numbers of fibers with CN and eMHC expression, and the persistent presence of fibers with CN which indicate prolonged muscle degeneration and do not complete differentiation into adult fibers. We also could not rule out the possibility of impaired neuromuscular transmission and disorganized neuromuscular junction (NMJ) structure in t/t muscle, which can be related to reduction of tetanic torque generation at high electrical frequency before and after the muscle injury [36, 37]. Elevated inflammatory cells (macrophage) in t/t muscle may also have destructive effects on the structure and function of motor neurons and the NMJ [38]. Collectively, these maladaptations demonstrate the compromised ability of skeletal muscles to regenerate and prolonged muscle degeneration in t/t mice during DCM induced HF.

In summary, skeletal muscles of mice with DCM-induced HF exhibit a unique myopathy characterized by decreased torque production, atrophy of oxidative fiber types, ongoing inflammation and repair under homeostasis, and impaired regeneration after acute muscle injury. This novel skeletal muscle myopathy likely contributes to and exacerbates exercise intolerance in DCM-induced HF.

## Additional file

Additional file 1: **Figure S1.** Loss of exercise capacity without change in hindlimb muscle mass. **Figure S2.** Altered torque-frequency relationship in t/t plantar flexors compared to WT. **Figure S3.** mRNA expression of inflammatory markers and myogenic regulators in t/t muscle. **Figure S4.** The number of satellite cells is not different in WT and t/t muscle. (DOCX 3542 kb)

## Abbreviations

CHF: Chronic heart failure, an end stage of heart failure when the heart is no longer able to pump blood adequately
cMyBP-C: Cardiac myosin binding protein-C, which is a sarcomeric thick filament regulatory protein
CN: Central nuclei, single nuclei positioned centrally in the cytosol
CSA: Cross-sectional area, the total area of the cross-sectioned muscle measured perpendicular to its long axis
DCM: Dilated cardiomyopathy, which is a disease in which the heart muscle becomes abnormally thin and ventricular chambers are dilated
FS (%): Percentage of fractional shortening, which is a measure of the heart’s muscular contractility and measured at the degree of shortening of the left ventricular diameter between end-diastole and end-systole
HCM: Hypertrophic cardiomyopathy, which is a disease in which the heart muscle becomes abnormally thick and ventricular chambers are constricted
HF: Heart failure, which is a condition when the heart cannot pump (systolic) or fill (diastolic) adequately
t/t: Cardiac myosin binding protein-C (cMyBP-C) null mouse model in which MYBPC3 gene was mutated to produce carboxy-terminal-truncated cMyBP-C, which would result in null phenotype and develop dilated cardiomyopathy
WT: Wild-type mouse; normal naïve mouse without any genetic alterations
