# Supplementary material for: Dilated cardiomyopathy-mediated heart failure induces a unique skeletal muscle myopathy with inflammation
Source: Skelet Muscle. 2019 Jan 24;9:4. doi: 10.1186/s13395-019-0189-y (PMC6345027; doi:10.1186/s13395-019-0189-y)
Supplement: Supplementary file 1 — Figure S1. Loss of exercise capacity without change in hindlimb muscle mass. Figure S2. Altered torque-frequency relationship in t/t plantar flexors compared to WT. Figure S3. mRNA expression of inflammatory markers and myogenic regulators in t/t muscle. Figure S4. The number of satellite cells is not different in WT and t/t muscle. (DOCX 3542 kb) [file 13395_2019_189_MOESM1_ESM.docx]

**Supplemental Figures**

**Dilated cardiomyopathy-mediated heart failure induces a unique skeletal muscle myopathy with inflammation**

Taejeong Song^1^, Palanikumar Manoharan^2^, Douglas P. Millay^3,4^, Sheryl E. Koch^1^, Jack Rubinstein^1^, Judith A Heiny^5^ and Sakthivel Sadayappan^1^*

^1^Heart Lung Vascular Institute, Division of Cardiology, University of Cincinnati, Cincinnati, OH 45267, USA

^2^Department of Molecular Genetics, Biochemistry, and Microbiology, University of Cincinnati, Cincinnati, OH 45267, USA

^3^Division of Molecular Cardiovascular Biology, Cincinnati Children's Hospital Medical Center, 240 Albert Sabin Way, Cincinnati, OH 45229, USA

^4^Department of Pediatrics, University of Cincinnati College of Medicine, Cincinnati, OH 45267, USA.

^5^Department of Pharmacology and Systems Physiology, University of Cincinnati, Cincinnati, OH 45267, USA

Running title: Skeletal muscle dysfunction in heart failure

*Address correspondence to Sakthivel Sadayappan, PhD, MBA, Department of Internal Medicine, Heart, Lung and Vascular Institute, Division of Cardiovascular Health and Sciences, College of Medicine, University of Cincinnati, 231 Albert Sabin Way, Cincinnati, OH 45267-0575, USA. Phone: +1 513-558-7498; Email: sadayasl@ucmail.uc.edu.

**
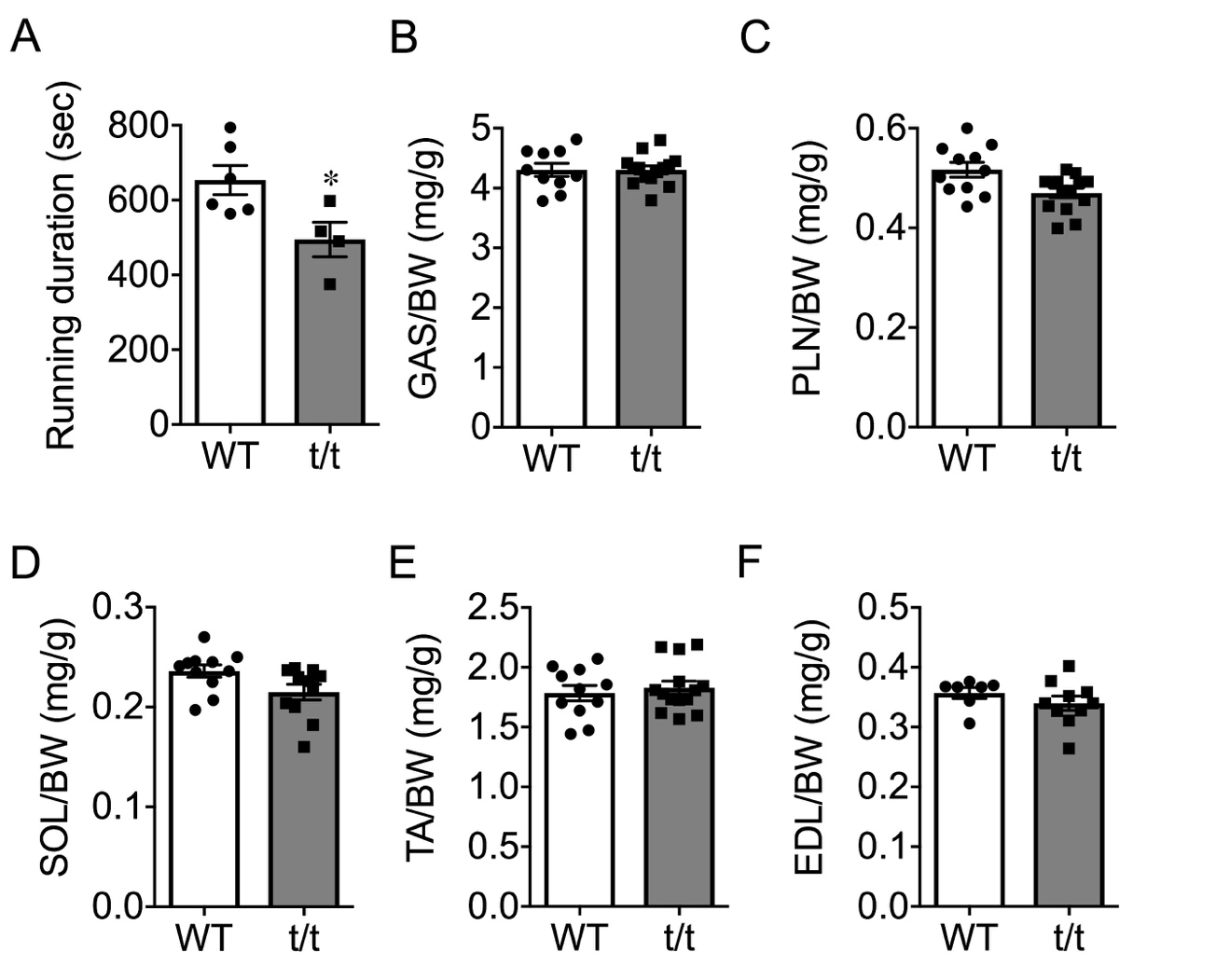
**

**Figure S1. Loss of exercise capacity without change in hindlimb muscle mass**. Total running duration (A) on the treadmill was measured during a graded maximum run test. Relative mass of five major hindlimb muscles,normalized to individual body mass: GAS (B), plantaris (PLN) (C), soleus (SOL) (D), tibialis anterior (TA) (E), and extensor digitorum longus (F) were compared between WT and t/t. *P<0.05.

**
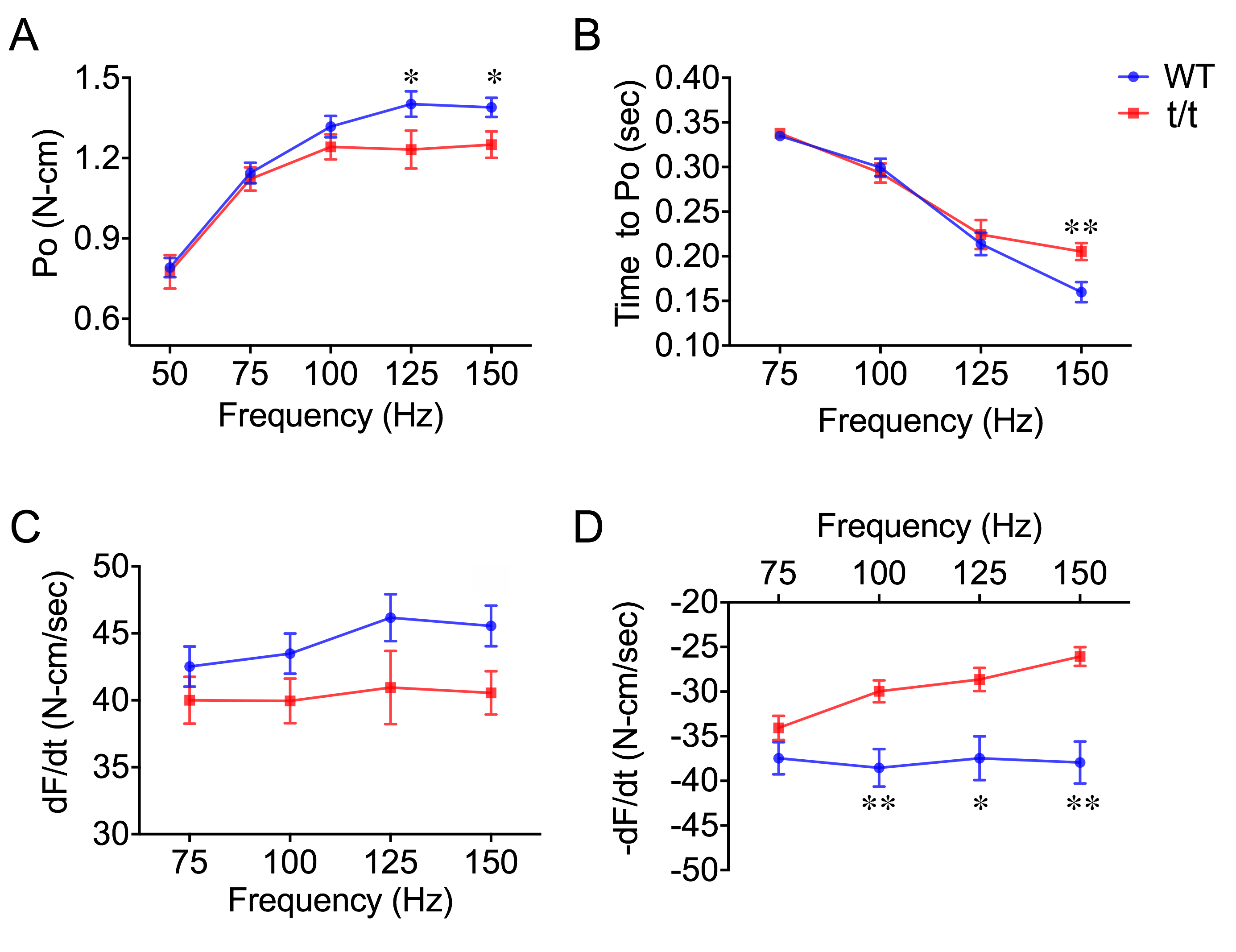
**

**Figure S2. Altered force-frequency relationship in t/t plantar flexors compared to WT.** A, Isometric tetanic tension (Po) was measured at as the frequency of tetanic stimulation was increased from 50 to 150Hz. B, Time-to-reach peak tetanic tension (Po) was recorded over the same range of stimulation frequencies . Rate-of-force development (C) and rate of relaxation (D) at each frequency were compared between groups. *P<0.05 and **P<0.01

**
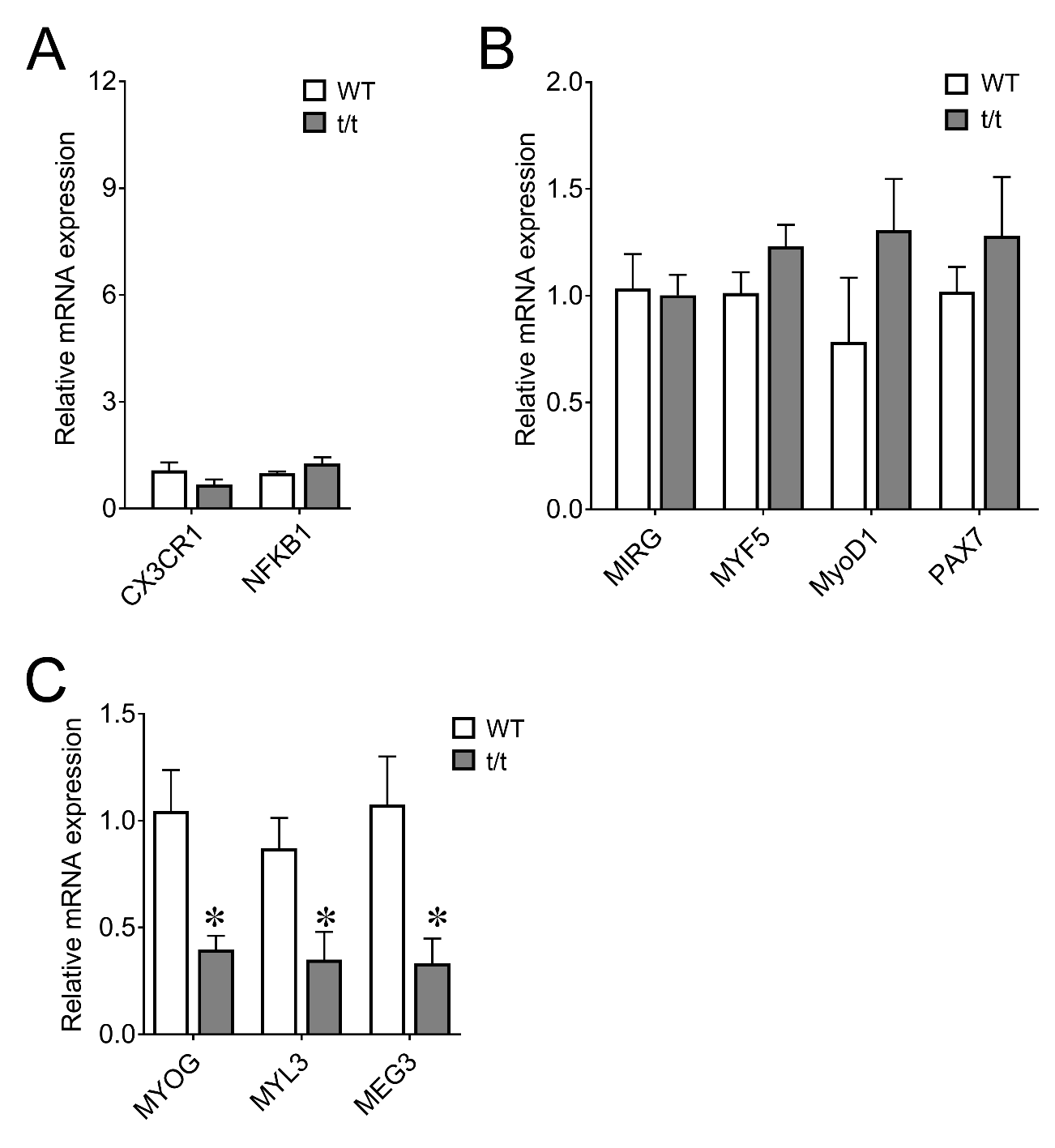
**

**Figure S3.** **mRNA expression of inflammatory markers and myogenic regulators in t/t muscle.** A. Gene expression of the chemokine receptor CX3CR1 and one key inflammatory transcription factor, NFKB1, did not change in HF. B. No significant differences of myogenic regulatory factors MIRG, MYF5, MyoD1, or PAX7 between t/t and WT (n=3-4 per group). C. Expression of MYOG, MYL3 and MEG3, key markers of myogenesis, significantly decreased in GAS of t/t compared to WT (n=5, P=0.018 and 0.026), suggesting incomplete regeneration.

**
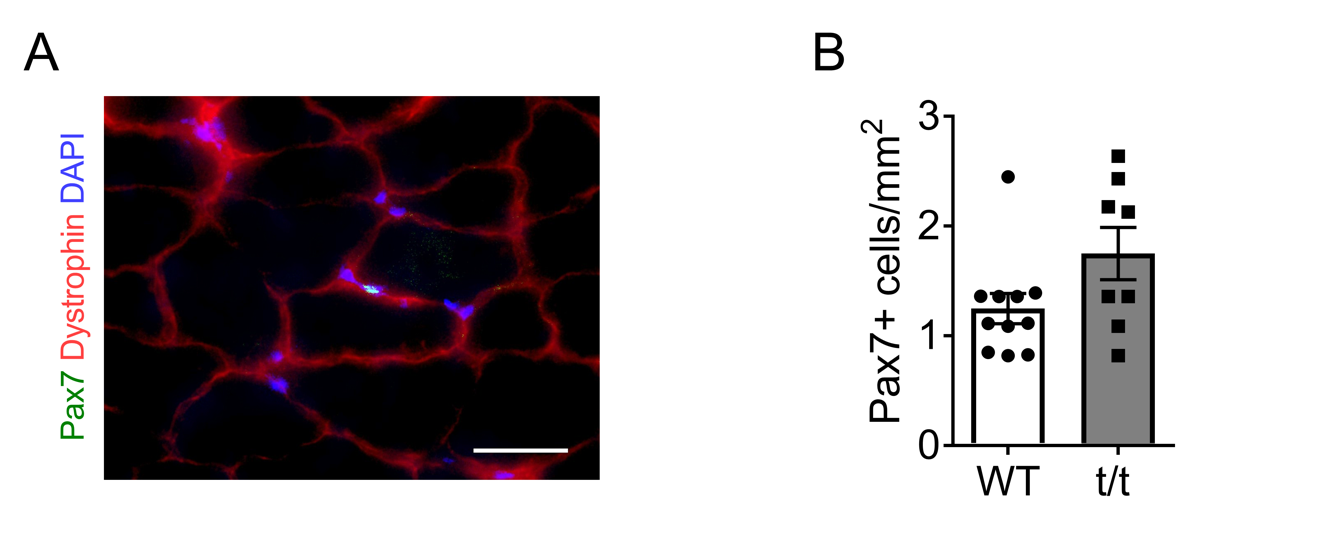
**

**Figure S4. The number of satellite cells is not different in WT and t/t muscle.** A. Representative image of Pax7 positive satellite cells. Scale bar: 50 µm. B. Average numbers of Pax7 positive satellite cells in cross-sectioned LG muscle.

Table S1. In vivo peak twitch torque (Pt)

|  | Pt (N-cm) | 1/2RT (ms) | dF/dt (N-cm/ms) | -dF/dt (N-cm/ms) | Time to Pt (ms) |
| --- | --- | --- | --- | --- | --- |
| WT | 0.33±0.02 | 13.75±0.6 | 37.41±1.36 | -18.59±1.08 | 17.75±0.4 |
| *t/t* | 0.31±0.02 | 12.44±0.7 | 36.23±1.61 | -18.79±1.32 | 16.75±0.3 |

Values are presented as mean ± SEM.
